# Supplementary material for: Antimicrobial Peptides in the Battle against Orthopedic Implant-Related Infections: A Review
Source: Pharmaceutics. 2021 Nov 12;13(11):1918. doi: 10.3390/pharmaceutics13111918 (PMC8625235; doi:10.3390/pharmaceutics13111918)
Supplement: Supplementary file 1 [file pharmaceutics-13-01918-s001.zip › pharmaceutics-1420971-supplementary.pdf]

pharmaceutics

MDPI

Supplementary materials: Antimicrobial Peptides in the Battle against Orthopedic Implant-Related Infections: A Review

Bruna Costa, Guillermo Martínez-de-Tejada, Paula A. C. Gomes, M. Cristina L. Martins * and Fabíola Costa

Table S1. Antimicrobial-peptides (AMPs) name and sequence used in the different works reviewed.

AMPs	Sequence	Refs
**hLF1-11**	GRRRRSVQWCA	[51-54, 126]
Modified hLF1-11	MPA^{δ}-PEG^{ϕ}-GRRRRSVQWCA-NH_2	[102]
Modified hLF1-11	MPA^{δ}-Ahx^{τ}-Ahx-Ahx-GRRRRSVQWCA-NH_2	[108]
Tet213	KRWWKWWRRC	[57,59, 64, 106]
HHC36	KRWWKWWRR	[59-61]
HHC36	KRWWKWWRR-NH_2	[56, 58]
Dhvar-5	LLLFLLKKRKKRKY	[49-50 127-128]
Halactine-2 analogues	I/H27: GKWMKLLKKILK-NH_2<br>II: gkwmkllkkilk-NH_2<br>III: GKWKKLLKKILK-NH_2<br>IV: GKWMKMLKKILK-NH_2<br>V: GKWLKLLKKILK-NH_2<br>VI/H39: GKWVKLLKKILK-NH_2<br>VII: gkwvkllkkilk-NH_2<br>VIII: GKWMKLLKKILK-NH_2<br>IX: KWMKLLKKILK-NH_2<br>X: kwmkllkkilk-NH2<br>XI: βA^{φ}KWMKLLKKILK-NH_2<br>XII: βakwmkllkkilk-NH_2<br>H27D: Gkwmkllkkilk- NH_2<br>H39D: Gkwvkllkkilk-NH_2<br>H39LD: GKwVkLiK*k*IiK-NH_2	[29, 55]
GL13K	GKIIKLKASLKLL-NH_2	[27, 104]
Modified GL13K	GKIIKLKASLKLL-$CONH_2$	[95, 96]
RRP9W4N	RRPRPRPRPWWWW-NH_2	[79]
Cateslytin	RSMRLSFRARGYGFR	[62]
AMP2	KRRWRIWLV –(CO)NH_2	[83]
β-peptide	$(ACHC^{\psi}$- β^3hV- β^3hK$)_3$	[32]
OP-145	Ac-IGKEFKRIVERIKRFLRELVRPLR-NH_2	[77]
SAAP-145	Ac-LKRLYKRLAKLIKRLYRYLKKPVR-NH_2	[63]
SAAP-276	Ac-LKRVWKAVFKLLKRYWRQLKKPVR-NH_2	[63]
LL-37	CLLGDFFRKSKEKIGKEFKRIVQRIKDFLRNLVPRTES	[98]
KR-12	KRIVQRIKDFLR-NH_2	[89]
FK-16	FKRIVQRIKDFLRNLV-NH_2	[99]
GZ3.163	4-methylhexanoyl-CdabDabDabLfDabDabL-NH_2	[97]
Melimine	CTLISWIKNKRKQRPRVSRRRRRRGGRRRR	[101]
JH8194	KRLFRRWQWRMKKY	[103, 113]
Cecropin B	KWKVFKKIEKMGRNIRNGIVKAGPAIAVLGEAKAL-NH_2	[120]
Bacitracin	CLeIlecy(LOrnIfHaA)	[105,121]
Chimeric peptides	LKLLKKLLKLLKKL	[111-112]
	KWKRWWWWR	[111]
Magainin I	GIGKFLHSAGKF-GKAFVGEIMKS	[116]
SYM11K	KKFPWWWPFKK	[117]
L_9K_6	LKLLKKLLKKLLKLL	
LASIO III	VNWKKILGKIIKVVK	

Tet20	KRWRIRVRVIRKC	[107]
HHC10-derivative	H-KRWWKWIRW-NH_2	[130]
-	CLATTLTIAT-NH_2	[125]
E14LKK	LKKLLKLLKKLLKL	[132]
Anoplin	GLLKRIKTLL	[134]
KSLW	KKVVFWVKFK	[115]
-	RRRRRRGALAGRRRRRRGALAGEEEEEEE	[139]

Note: d-amino acids are shown in lower case letters; $^{\delta}$MPA, 3-mercaptopropionic acid; $^{\phi}$PEG, 2 units of 8-amino-3,6-dioxaoctanoic acid; $^{\tau}$6-aminohexanoic acid; $^{\varphi}\beta$A, beta-alanine; $^{\psi}$ aminocyclohexanecarboxylic acid.
